# Supplementary material for: MAOA uVNTR Genetic Variant and Major Depressive Disorder: A Systematic Review
Source: Cells. 2022 Oct 17;11(20):3267. doi: 10.3390/cells11203267 (PMC9600429; doi:10.3390/cells11203267)
Supplement: Supplementary file 1 [file cells-11-03267-s001.zip › cells-1835957-supplementary.pdf]

cells MDPI

Review

MAOA uVNTR genetic variant and Major Depressive Disorder: A Systematic Review

Ana Beatriz Castro Gonçalves[1], Caroline Ferreira Fratelli[2,3], Jhon Willatan Saraiva Siqueira[2], Ligia Canongia de Abreu Cardoso Duarte[2], Aline Ribeiro Barros[2], Isabella Possatti[2], Maurício Lima dos Santos[4], Calliandra Maria de Souza Silva[4], and Izabel Cristina Rodrigues da Silva[4*]

[1] Undergraduate Student, Farmacy Course, Faculty of Ceilândia, University of Brasília (UnB), DF, Brazil
[2] Postgraduate Program in Health Sciences and Technologies, Faculty of Ceilândia, University of Brasília (UnB), DF, Brazil
[3] Faculty of Higher Education of the Reunited Amazon (FESAR), PA, Brazil.
[4] University of Brasília (UnB), DF, Brazil

* Correspondence: Dra. Izabel Cristina Rodrigues da Silva. Campus Universitário, s/n, Centro Metropolitano, Brasília-DF, 72220-275. (61) 3107-8400 belbiomedica@gmail.com

Supplementary Material

Table S1: Articles excluded according to criteria related to the defined PECOS strategy.

Author	Year	Article's Title	Reason for Exclusion	Exclusion Step
Ma, et al.	2013	Association between MAOA-u VNTR polymorphism and its interaction with stressful life events and major depressive disorder in adolescents	Duplicate	Title and abstract analysis
Lung, et al.	2011	Association of the MAOA promoter uVNTR polymorphism with suicide attempts in patients with major depressive disorder	Duplicate	Title and abstract analysis

Du, et al.	2004	MAO-A gene polymorphisms are associated with major depression and sleep disturbance in males	Duplicate	Title and abstract analysis
Fan, et al.	2010	Meta-analysis of the association between the monoamine oxidase-A gene and mood disorders	Duplicate	Title and abstract analysis
Rahikainen, et al.	2017	Serotonergic 5HTTLPR/rs25531 s-allele homozygosity associates with violent suicides in male citalopram users	Duplicate	Title and abstract analysis
Hung, et al.	2012	Monoamine oxidase A gene polymorphism and suicide: an association study and meta-analysis	Duplicate	Title and abstract analysis
Kinnally, et al.	2009	Parental care moderates the influence of MAOA-uVNTR genotype and childhood stressors on trait impulsivity and aggression in adult women	Duplicate	Title and abstract analysis
Dannlowski, et al.	2009	Reduced amygdala–prefrontal coupling in major depression: association with MAOA genotype and illness severity	Duplicate	Title and abstract analysis
Huang, et al.	2009	Association of monoamine oxidase A (MAOA) polymorphisms and clinical subgroups of major depressive disorders in the Han Chinese population	Duplicate	Title and abstract analysis
Sanabrais-Jiménez, et al.	2022	Association study of Catechol-O-Methyltransferase (COMT) rs4680 Val158Met gene polymorphism and suicide attempt in Mexican adolescents with major depressive disorder	Duplicate	Title and abstract analysis
Yu, et al.	2005	Association study of a monoamine oxidase a gene promoter polymorphism with major depressive disorder and antidepressant response	Duplicate	Title and abstract analysis

Brummett, et al.	2007	Associations of a regulatory polymorphism of monoamine oxidase-A gene promoter (MAOA-uVNTR) with symptoms of depression and sleep quality	Study population	Title and abstract analysis
Fan, et al.	2010	Meta-analysis of the association between the monoamine oxidase-A gene and mood disorders	Study type	Title and abstract analysis
Huang, et al.	2008	Neither single-marker nor haplotype analyses support an association between monoamine oxidase A gene and bipolar disorder	Study population	Title and abstract analysis
Hung, et al.	2012	Monoamine oxidase A gene polymorphism and suicide: an association study and meta-analysis	Study type	Title and abstract analysis
Lee, et al.	2010	MAOA interacts with the ALDH2 gene in anxiety-depression alcohol dependence	Study population	Title and abstract analysis
Miller, et al.	2010	Acute intravenous synaptamine complex variant KB220™"normalizes" neurological dysregulation in patients during protracted abstinence from alcohol and opiates as observed using quantitative electroencephalographic and genetic analysis for reward polymorphisms: Part 1, pilot study with 2 case reports	Study population	Title and abstract analysis
Phillips-Bute, et al.	2008	Relationship of genetic variability and depressive symptoms to adverse events after coronary artery bypass graft surgery	Study population	Title and abstract analysis
Author	Year	Article's Title	Reason for Exclusion	Exclusion Step

Rahikainen, et al.	2017	Serotonergic 5HTTLPR/rs25531 s-allele homozygosity associates with violent suicides in male citalopram users	Study population	Title and abstract analysis
Wang, et al.	2007	Possible interaction between MAOA and DRD2 genes associated with antisocial alcoholism among Han Chinese men in Taiwan	Study type	Title and abstract analysis
Cusin, et al.	2002	Association study of MAO-A, COMT, 5-HT2A, DRD2, and DRD4 polymorphisms with illness time course in mood disorders	Study population	Full text analysis
Sabol, et al.	1998	A functional polymorphism in the monoamine oxidase A gene promoter	Study population	Full text analysis
Christiansen, et al.	2007	Candidate gene polymorphisms in the serotonergic pathway: influence on depression symptomatology in an elderly population	Study population	Full text analysis
Serretti, et al.	2002	Family-based association study of 5-HTTLPR, PH, MAO-A, and DRD4 Polymorphisms in mood disorders	Study population	Full text analysis
Muramatsu, et al.	1997	Monoamine oxidase genes polymorphisms and mood disorder	Incomplete Data	Full text analysis
Furlong, et al.	1999	Analysis of the monoamine oxidase A (MAOA) gene in bipolar affective disorder by association studies, meta-analyses, and sequencing of the promoter	Study type	Full text analysis
Author	Year	Article's Title	Reason for Exclusion	Exclusion Step

Ma, et al.	2013	Association between MAOA-u VNTR polymorphism and its interaction with stressful life events and major depressive disorder in adolescents	Language of article (in Chinese)	Full text analysis
Kinnally, et al.	2009	Parental care moderates the influence of MAOA-uVNTR genotype and childhood stressors on trait impulsivity and aggression in adult women	Study population	Full text analysis
Dannlowski, et al.	2009	Reduced amygdala–prefrontal coupling in major depression: association with MAOA genotype and illness severity	Incomplete data	Full text analysis
Dannlowski, et al.	2009	MAOA genotype: Impact on cortico-limbic functional connectivity in major depression	Study type	Full text analysis

Source: Own authorship.

Table S2: Quality Evaluation of articles according to the adapted GRIPS guideline.

Section/Topic		Huang et al, 2009 (1)	Lung et al, 2011 (2)	Yu et al, 2005 (3)	Rivera et al, 2009 (4)	Du et al, 2004 (5)	Won et al, 2016 (6)	Sanabrais-Jiménez et al, 2021 (7)
**Methods**								
**Study design and setting**	4) Present key elements of study design early in the paper and describe the setting, locations, and relevant dates, including periods of recruitment, exposure, follow-up, and data collection.	x	x	x	x	x	x	x
**Participants**	5) Describe eligibility criteria for participants, and sources and methods of selection of participants.	x	x	x	x	x	x	x
**Variables: Definition**	6) Clearly define all participant characteristics, risk factors and outcomes. Clearly define genetic variants using a widely-used nomenclature system.	x	x	x	x	x	x	x
**Variables: Assessment**	7) (a) Describe sources of data and details of methods of assessment (measurement) for each variable.	x	x	x	x	x	x	x
	(b) Give a detailed description of genotyping and other laboratory methods.	x	x	x	x	x	x	x
**Variables: Coding**	8) (a) Describe how genetic variants were handled in the analyses	x	x	x	x	x	x	x
	(b) Explain how other quantitative variables were handled in the analyses. If applicable, describe which groupings were chosen, and why.	x	x	x	x	x	x	x
**Analysis: Risk model construction**	9) Specify the procedure and data used for the derivation of the risk model. Specify which candidate variables were initially examined or considered for inclusion in models. Include details of any variable selection procedures and other model building issues. Specify the horizon of risk prediction (e.g., 5-year risk).		x		x			
**Analysis: Validation**	10) Specify the procedure and data used for the validation of the risk model.		x		x			
**Analysis: Missing data**	11) Specify how missing data were handled.							

**Analysis: Statistical methods**	12) Specify all measures used for the evaluation of the risk model including, but not limited to, measures of model fit and predictive ability.	x	x	x	x	x	x	x
**Analysis: Other**	13) Describe all subgroups, interactions, and exploratory analyses that were examined.	x	x	x	x	x	x	x
**Results**								
**Participants**	14) Report the numbers of individuals at each stage of the study. Give reasons for nonparticipation at each stage. Report the number of participants not genotyped, and reasons why they were not genotyped.	x	x	x	x	x	x	x
**Descriptives: Population**	15) Report demographic and clinical characteristics of the study population, including risk factors used in the risk modeling.		x	x	x	x	x	x
**Descriptives: Model estimates**	16) Report unadjusted associations between the variables in the risk model(s) and the outcome. Report adjusted estimates and their precision from the full risk model(s) for each variable.	x	x	x	x	x	x	
**Risk distributions**	17) Reports the predicted risk distribution and/or its scores.	x	x	x	x	x		
**Assessment**	18) Report measures of model fit and predictive ability, and any other performance measures, if pertinent.	x	x		x			
**Validation**	19) Report any validation of the risk model(s).	x	x	x	x			
**Other analyses**	20) Present results of any subgroup, interaction, or exploratory analyses, whenever pertinent.	x	x	x	x	x	x	x
**Discussion**								
**Limitations**	21) Discuss limitations and assumptions of the study, particularly those concerning study design, selection of participants, and measurements and analyses, and discuss their impact on the results of the study.		x	x		x	x	x

**Interpretation**	22) Give an overall interpretation of results considering objectives, limitations, multiplicity of analyses, results from similar studies, and other relevant evidence.	x	x	x	x	x	x	x
**Generalizability**	23) Discuss the generalizability and, if pertinent, the health care relevance of the study results.	x	x	x	x	x	x	x

x = Present.

Table 3S: The selected articles' selection criteria (MDD and control groups), ethnicity/race, and employed statistical analysis per their original authors, with few modifications.

Articles	*Selection Criteria and Study's Ethnicity*		Statistical Analysis Employed
	**MDD group**	**Control group**	
Yu et al. 2005 (China) (3)	Psychiatric outpatients were included if they met the Diagnostic and Statistical Manual of the American Psychiatric Association (DSM-IV) criteria for MDD and a minimum baseline score of 18 on the 21-item Hamilton Depression Rating Scale (HAM-D) (8). They also had to have the presence of depressive symptoms for at least two weeks before entry into the study without antidepressant treatment (patients were new cases or had quit antidepressant treatment for more than two weeks).<br>Exclusion criteria were additional diagnoses on Axis I (including substance abuse, generalized anxiety disorders, panic disorders, or obsessive-compulsive disorders) of the DSM-IV, personality disorders, pregnancy, attempted suicide, major medical/neurological disorders, and bipolar depressive patients.	Most of the healthy controls were medical staff, and they were each interviewed to rule out psychotic or mood disorders.	All MDD patients took fluoxetine (range: 20–40 mg/ day; mean 25.7 (9.0) mg/day), and treatment efficacy was evaluated by determining HAM-D before and after the 4-week fluoxetine treatment by an expert in psychiatric rating, blinded to the patient's genotype. Therapeutic response was assessed by the percentage score reduction in HAM-D scores [(baseline score - 4-week score) x 100/baseline score].<br>The categorical data were analyzed using the Chi-square (X^2) or Fisher's exact test, as appropriate. Differences in continuous variables were evaluated by Student's t-test (e.g., the age difference between the MDD and control; comparison of the HAM-D change after fluoxetine treatment between two genotypic groups in MDD male) or one-way analysis of variance followed by the LSD multiple range tests for comparison among groups (e.g., comparison of the HAM-D change after fluoxetine treatment among three genotypic groups in MDD female). The criterion for significance was set at $p < 0.050$ for all tests. Data are presented as mean (SD).<br>The 3.5R allele frequency was zero; in addition, the 2R and 5R alleles were rare in this study. Thus, the six individuals who carried 2R or 5R alleles were excluded from the statistical analyses (thus, MDD = 228 and Control = 213).
	The sample (controls and MDD patients) consisted entirely of ethnic Han Chinese. Age range 18–79 years.<br>This study was an add-on to another research project (9).		
Huang et al. 2009 (Taiwan) (1)	The MDD group was recruited either in their first episode or in a recurrent episode from clinical settings and evaluated using the Chinese Version of the Schedule of Affective Disorder and Schizophrenia-Lifetime (SADS-L) (10) and DSM-IV criteria (11). All patients in this study met DSM-IV criteria for MDD with a minimum score of 18 on the HAM-D. Individuals with a history of substance dependence, severe medical illness, organic brain disease, or any concomitant major psychiatric disorders were excluded. The patients were further classified into four clinical subgroups: MDD with a family history (one or	The control group included healthy volunteers recruited from the community, and the Chinese Version of SADS-L was used to screen out psychiatric conditions. Control subjects were free of past/present major/minor mental illness, including affective disorder, schizophrenia, anxiety disorder, personality disorder, and substance use disorders, and those with a family history of such disorders were also excluded.	Independent-samples t-tests were employed to determine differences in mean age between patients with MDD and healthy controls. Pearson's Chi-square (X^2) analysis compared gender differences between the patient and control groups. Hardy-Weinberg equilibrium was assessed for each group, and the genotype and allele frequencies were also compared in patients versus controls using Pearson X^2 analyses. Fisher exact tests were substituted for the Pearson X^2 test when sample sizes were smaller than expected (less than five subjects). Multiple logistic regression analysis was applied to correct the effects of possible covariates such

	more first-degree relatives had either bipolar disorder or MDD), MDD without a family history, moderate MDD (18 ≤ HAM-D ≤ 24), and severe MDD (HAMD > 24).		as age, gender, and other MAOA polymorphisms on the risk of MDD. SPSS (version 11.5, SPSS, Taipei, Taiwan) statistical software was used for all analyses, and a probability value of $P<0.05$ was considered statistically significant.<br>Haplotype frequencies, linkage disequilibrium coefficients, and standardized linkage disequilibrium coefficients between the MAOA gene's *uVNTR* and *EcoRV*[b] polymorphisms were estimated using two computer programs: (1) estimating haplotypes and (2) permutation and model-free analysis. The haplotype frequency examination used Fisher's exact test when small cell sizes were encountered (12). Power analyses were performed using G-Power computer software, and effect size conventions were determined according to Erdfelder et al. (13) method. All tests were two-tailed with the α set at 0.05.<br>The 5R allele was not found, and the 2R allele was rare in this study; therefore, subjects with the 2R allele were excluded from the statistical analyses. For haplotype data analysis (between *MAOA-uVNTR* and *MAOA-EcoRV*[b]) and single *MAOA-uVNTR*, the study included 277 MDD patients (107 males, 170 females) and 308 healthy controls (197 males, 111 females).
	The sample (controls and MDD patients) consisted entirely of the Han Chinese population in northern Taiwan. All participants were unrelated and matched for ethnicity and geographic origin.		

<table>
<tr><td rowspan="2">Lung et al. 2011 (Taiwan) (2)</td><td>The study recruited four groups of participants: 1) healthy controls from the community, 2) patients with MDD - recruited from a teaching hospital in southern Taiwan from April 2001 to March 2006, 3) subjects without mental disorders who had attempted suicide recruited from the emergency room, and 4) patients with MDD who had attempted suicide.
Two senior psychiatrists and research assistants interviewed all the participants to ensure that they did or did not meet the psychiatric diagnosis of MDD, according to the Diagnostic and Statistical Manual of Mental Disorders (11) using the Mini-international Neuropsychiatric Interview (MINI) (14).
Personality (assessed by the Eysenck Personality Questionnaire (EPQ), a 25-item self-report inventory measuring the personality traits of extraversion and neuroticism) and symptom (assessed using the Chinese Health Questionnaire (CHQ), a 12-item screening instrument used to identify minor psychiatric disorders in individuals in the community or nonpsychiatric departments) profiles were collected.</td><td>Not stated explicitly (see MDD group).</td><td rowspan="2">In the descriptive analysis, continuous variables were expressed as the mean ± SD, and categorical variables were shown as proportions. The study used the Guo and Thompson (15) algorithm, which allows an exact test for traits encoded by multiple alleles, to test whether the <i>MAOA uVNTR</i> variant's allelic frequency in females was in Hardy-Weinberg equilibrium. Pearson's chi-square (X^2) test was applied to distinguish differences among the four groups studied. Moreover, the allelic variants were categorized further into two groups according to their transcriptional activity, as described by Sabol et al. (16). Genotypes homozygous/hemizygous[a] for the 3R allele formed the low-activity group, whereas those homozygous/hemizygous[a] for the 4R allele formed the high-activity group. Pearson X^2 test was also used to determine whether intergroup differences in allelic frequency between the 3R and 4R variants were significant.
Binary logistic regression analysis was conducted to clarify which factors among <i>MAOA uVNTR</i> variant, age, personality traits, gender, smoking, parental attachment, and mental health condition were associated with either suicide attempts in patients with MDD or depressive symptoms in individuals who had attempted suicide. P-values <0.05 were considered statistically significant. These statistical analyses were carried out using the SPSS 17.0 software for Windows. Structural Equation Modelling (SEM) was performed using the AMOS 7.0 software for Windows to illustrate the interrelationships between the variables studied, which included personality scores, depression, anxiety, suicidal behavior, MDD, and the <i>MAOA uVNTR</i> 3R allele presence. The criteria that indicated that the null hypothesis model corresponded to the true structure were p-values >0.05 and adjusted goodness-of-fit index (AGFI) >0.9.
The 2R and 3R allelic variants were defined as short-form variants, and the remaining variants, with a repeat number greater than three, were defined as long-form variants. The R2 and R5 allele frequencies were ignored from the statistical analyses because they were low in the studied population.</td></tr>
<tr><td colspan="2">The sample (controls and MDD patients) is from southern Taiwan.</td></tr>
<tr><td>Won et al. 2016 (Korea) (6)</td><td>Medication-naïve female patients with MDD were recruited from the outpatient psychiatric clinic of Korea University Anam Hospital in Seoul, Republic of Korea. A board-certified</td><td>Age, sex, and education level matched healthy controls were recruited by advertisements from the community and screened for significant psychiatric</td><td>According to genotype, patients and controls were divided into two subgroups: MAOA-uVNTR high-activity allele carriers (MAOA-H: 3R/4R and 4R/4R) versus MAOA-uVNTR low-activity allele</td></tr>
</table>

<table>
<tr>
<td rowspan="2"></td>
<td>psychiatrist determined the diagnosis according to the Diagnostic and Statistical Manual for Mental Disorders-IV-Text Revision (DSM-IV-TR), using the Korean version of the Structured Clinical Interview for DSM-IV. The depression severity was measured by the 17-item Hamilton Depression Rating Scale (HRDS) on the day of magnetic resonance imaging (MRI) acquisition. Patients with primary or comorbid psychiatric diagnoses other than MDD and patients suffering from serious or unstable medical illness and primary neurological illness were excluded from the study.</td>
<td>histories. None had a psychiatric disorder.</td>
<td rowspan="2">carriers (MAOA-L: 2R/3R and 3R/3R). Although heterozygous females with one or two low-activity alleles have been grouped as MAOA-L in some studies, others have grouped such subjects as MAOA-H [9], similar to this study's subgrouping.
Differences in demographic and clinical characteristics between MDD patients and controls were analyzed using one-way ANOVA for continuous variables (age, years of education, illness duration, and HDRS scores). Hardy–Weinberg equilibrium (HWE) of MAOA-uVNTR allele frequencies was analyzed using a Chi-square (X^2) test.
The difference in averaged cortical thickness (mm) of the OFC, including the medial and lateral sub-regions, between MDD patients and controls was analyzed using ANCOVA with age as a covariate. When comparing the OFC as a whole, the right and left OFC were separately tested, at $p < 0.05/2 = 0.025$, for multiple comparisons with Bonferroni correction. When comparing the sub-regions of the OFC, 4 sub-regions (left medial OFC, left lateral OFC, right medial OFC, and right lateral OFC) were separately tested at $p < 0.05/4 = 0.0125$ after Bonferroni correction. The interaction of diagnosis (MDD patients, healthy controls) and MAOA-uVNTR genotype (MAOA-H, MAOA-L) on OFC thickness was examined using ANCOVA with age as a covariate.</td>
</tr>
<tr>
<td colspan="2">All subjects (controls and MDD patients) were right-handed (revealed by the Edinburgh Handedness Test) and were self-identified Koreans with ethnic origin ascertained by confirming the ethnicity of three generations of the patient's families. Age range 23–60 years.</td>
</tr>
</table>

Du et al. 2004 (Canada) (5)	Unrelated patients suffering major depressive disorder (either first or recurrent episode) attending the Royal Ottawa Hospital Psychopharmacology Unit for drug trials of new antidepressant medications participated in the study. Only subjects with a minimum score of 18 on HAMD (17 items) and no prior (<2 weeks) antidepressant treatment were included in this study, and all patients were free of any other axis I or axis II diagnosis, including other mood or anxiety disorders, schizophrenia, alcohol or drug abuse. The other exclusion criteria included epilepsy, psychotic symptoms, significant organic brain disease, and clinically significant somatic diseases.	Unrelated control subjects, recruited by local advertising and screened for apparent medical or psychiatric illness, included in the study had no history of serious medical or mental illness, were drug-free, had no family history of psychiatric illness, and had a self-rating Beck's Depression Inventory (BDI) score of <7 (mean±BDI score: 1.0±1.6).	Allele and genotype frequencies between MDD patients and controls were compared by an X^2 test (two-tailed) and, where appropriate, by odds ratios with confidence intervals. Departure from Hardy-Weinberg equilibrium was calculated from genotype frequencies and tested by the X^2 goodness of fit test. Clusters of Hamilton Depression Rating Scale (HAMD) items were computed for each patient and analyzed separately from total HAMD scores, as described elsewhere (17). The differences in the HAM-D clusters' mean scores were compared by unpaired t-test (SPSS 9.0, SPSS Inc., Chicago). Since the *MAO-A* gene is X-chromosome linked, the data for males and females were analyzed separately, with Bonferroni correction being used to account for multiple testing.<br>With the study's sample size, a difference of ≥ 10% in allele frequency would be detected, with a power of 80% and α = 0.05 (SamplePower, SPSS Inc., Chicago). In the analysis, the 3R allele was denoted as allele 1 (less active) and grouped 3.5R, 4R, and 5R alleles as allele 2 (more active). Haplotype frequencies were estimated using the Estimating Haplotypes program, which uses the gene counting method to provide maximum-likelihood estimates of haplotype frequencies. The 2LD program evaluated the linkage disequilibrium.
	The sample (controls and MDD patients) was drawn from the same population pool (Ottawa area). Age range ~ 23–54 years. The sample composition was approximately 96.7% (%mean) Caucasian.		
Sanabrais-Jiménez et al. 2021 (Mexico) (7)	Unrelated Mexican adolescents who met DSM-IV-TR criteria for MDD and had a suicide attempt (SA) in the last 6 months were recruited in the inpatient and outpatient services from the Hospital Psiquiatrico Infantil 'Juan N. Navarro'. Of these patients, 63 have had a history of at least one SA and 134 without SA.<br>Patients were evaluated with the diagnostic interview semi-structured Schedule for Affective Disorders and Schizophrenia for School-Age Children-Present and Lifetime version (K-SADS-S-PL) (18). This interview confirmed the depression diagnosis and determined the type and number of comorbid diagnoses and the SA occurrence of the current depressive episode.<br>SA Assessment was evaluated by the five items concerning suicidal ideation and behavior during the screening interview of the K-SADS-PL and was only considered SA when all the	Not included in the study.	Demographics and clinical characteristics were analyzed using Chi-square (X^2) tests and t-student tests using the program RStudio version 1.0.136 (19). Genotype and allele distributions were analyzed using X^2 tests, with Bonferroni correction for multiple testing applied (five polymorphisms corrected at $p < 0.01$). The odds ratio was obtained using the 'epitools' version 0.5–10.1 package (20). The power analysis was performed using the R's 'gap' version 1.2.2 package (21) and showed, for the sample, a power of 0.88, assuming an additive genetic model, a risk allele frequency of 0.35, a population prevalence of SA in adolescents of 2.7%, an α level of 0.05 and a portion of cases of 0.31 in a sample of 197 patients.

	informants (patients and parents) agreed on the intention of dying.<br><br>All participants were Mexican Mestizos (mixed race, especially in this case having Spanish and indigenous descent) with a family background of three generations born in Mexico. Age range 12 -17 years.	
Rivera et al. 2009 (Spain) (4)	This study is a case-control PREDICT-Gene study with a Spanish sample of primary care patients nested in the PREDICT study of depression, a prospective study aiming to identify predictors of depression onset in primary care (22,23).<br>Using the EPI Info STATCAL power facility, the sample size needed to test a potential association between *MAOA-uVNTR* high-activity allele carriers and depression was estimated. The calculations were based on the following assumptions: (a) reported frequencies for *MAOA-uVNTR* high-activity allele carriers (77%) in the Spanish population (24); (b) reported prevalences of DSM-IV major depression (20%) in Spanish primary care samples (25); and, (c) the most conservative effect size reported (OR = 1.74), as described elsewhere (3). The final sample size of 1,228 subjects (242 DSMIV depressed cases and 980 controls) exceeds the needed sample of 1,185 (237 DSMIV cases and 948 controls) calculated on the above grounds.<br><br>All consecutive attendees to participating primary care practices in the areas of Malaga and Granada (Spain) were invited to take part in the genetic study. Data collection was completed between discrete periods starting in April 2004 and ending in December 2007. Participants aged over 75, unable to understand Spanish, and those with an organic mental disorder and/or any terminal illness were excluded.	Alleles were classified into two groups, low-activity alleles (3R allele) and high-activity alleles (3.5R, 4R, or 5R alleles), and the participants were also divided depending on their genotypes: homozygous for low-activity alleles (3R/3R) carriers and high-activity alleles carriers (i.e., either homozygous (3.5R/3.5R; 4R/4R; 5R/5R) or heterozygous for high-activity alleles).<br><br>Independent Variables<br>The PREDICT risk factor assessment was shown to have adequate test-retest reliability (23). Accurate information was gathered on socio-demographic data such as sex, age, education, marital status, living arrangements, and profession. To measure these factors, the study used either previously validated measures or subjected new measures to reliability testing at the outset of the study, as described elsewhere (23).<br><br>Dependent Variables: Measures of Depression<br>The depression section of the Composite International Diagnostic Interview (CIDI) was used to ascertain the diagnoses of ICD-10 Depressive Episode (mild, moderate, or severe) and DSM-IV Major Depression. The CIDI was administered by trained lay interviewers. The study used three different depressive outcomes: ICD-10 Depressive Episode (ICD-10 DE), ICD-10 Severe Depressive Episode (ICD-10 SDE), and DSM-IV Major Depression (DSM-IV MD).<br><br>Statistical Analysis<br>Analyses were performed using the SPSS 15.0 statistical package. The study tested all associations using three different depressive outcomes (ICD-10 DE, ICD-10 SDE, and DSM-IV MD). Analyses using ICD-10 SDE as an outcome excluded mild or moderate depression cases. As the *MAOA-uVNTR variant* is on the X chromosome, the study statistically explored all associations in the

		whole sample and among men or women separately. Chi-square (X2) tests were used to compare genotype and allele frequencies between those with and without depressive outcomes. Odds ratios with 95% confidence intervals were calculated using binary logistic regression analyses to compare both allele or genotype frequencies among patients and controls. The study successively analyzed the latter associations using all three depressive outcomes, first in women, then in men, and finally, in the whole sample. Crude and adjusted (by age/sex as appropriate) odds ratios were calculated for all the above regression analyses.

Note: [a]. *MAOA* is an X-linked gene; therefore, males are considered hemizygotes (have only one allele, e.g., A* or B*, with * indicating the missing chromosome) for the gene. Females can be homozygotes (have two of the same allele, e.g., A/A) or heterozygotes (have two different alleles, e.g., A/B) for the gene.

[b]. MAOA *EcoRV* (rs1137070; 1460C>T) genetic variant affects its enzyme activity, and its alleles are differentiated by the absence (-; C) or presence (+; T) of the *EcoRV* restriction length polymorphism site on exon 14 (position 1460).

References

1. Huang SY, Lin MT, Lin WW, Huang CC, Shy MJ, Lu RB. Association of monoamine oxidase A (MAOA) polymorphisms and clinical subgroups of major depressive disorders in the Han Chinese population. The World Journal of Biological Psychiatry. 2009 Jan 8;10(4–2):544–51.
2. Lung FW, Tzeng DS, Huang MF, Lee MB. Association of the MAOA promoter uVNTR polymorphism with suicide attempts in patients with major depressive disorder. BMC Medical Genetics. 2011 Dec 24;12(1):74.
3. Yu YWY, Tsai SJ, Hong CJ, Chen TJ, Chen MC, Yang CW. Association Study of a Monoamine Oxidase A Gene Promoter Polymorphism with Major Depressive Disorder and Antidepressant Response. Neuropsychopharmacology. 2005 Sep 1;30(9):1719–23.
4. Rivera M, Gutierrez B, Molina E, Torres-Gonzalez F, Bellon JA, Moreno-Kustner B, et al. High-Activity Variants of the uMAOA Polymorphism Increase the Risk for Depression in a Large Primary Care Sample. American journal of medical genetics part b-neuropsychiatric genetics. 2009;150(3):395–402.
5. Du L, Bakish D, Ravindran A, Hrdina PD. MAO-A gene polymorphisms are associated with major depression and sleep disturbance in males. NeuroReport. 2004 Sep;15(13):2097–101.
6. Won E, Choi S, Kang J, Lee MS, Ham BJ. Regional cortical thinning of the orbitofrontal cortex in medication-naïve female patients with major depressive disorder is not associated with MAOA-uVNTR polymorphism. Annals of General Psychiatry. 2016 Dec 12;15(1):26.
7. Sanabrais-Jiménez MA, Aguilar-García A, Hernández-Muñoz S, Sarmiento E, Ulloa RE, Jiménez-Anguiano A, et al. Association study of Catechol-O-Methyltransferase (COMT) rs4680 Val158Met gene polymorphism and suicide attempt in Mexican adolescents with major depressive disorder. Nordic Journal of Psychiatry [Internet]. 2021 Aug 3;1–5. Available from: https://doi.org/10.1080/08039488.2021.1945682
8. Sabol SZ, Hu S, Hamer D. A functional polymorphism in the monoamine oxidase A gene promoter. Human genetics. 1998 Sep;103(3):273-9.
9. Gutiérrez B, Arias B, Gastó C, Catalán R, Papiol S, Pintor L, Fañanás L. Association analysis between a functional polymorphism in the monoamine oxidase A gene promoter and severe mood disorders. Psychiatric genetics. 2004 Dec 1;14(4):203-8.
